# Pain sensitivity and analgesic use among 10,486 adults: the Tromsø study

**DOI:** 10.1186/s40360-017-0149-2

**Published:** 2017-06-09

**Authors:** Per-Jostein Samuelsen, Christopher Sivert Nielsen, Tom Wilsgaard, Audun Stubhaug, Kristian Svendsen, Anne Elise Eggen

**Affiliations:** 1 Regional Medicines Information and Pharmacovigilance Centre (RELIS), University Hospital of North Norway, P.O. Box 79, N-9038, Tromsø, Norway; 2 Department of Community Medicine, UiT The Arctic University of Norway, Tromsø, Norway; 3 Division of Mental Health, Norwegian Institute of Public Health, Oslo, Norway; 4 Department of Pain Management and Research, Division of Emergencies and Intensive Care, Oslo University Hospital, Oslo, Norway; 5 Institute of Clinical Medicine, Faculty of Medicine, University of Oslo, Oslo, Norway; 6 Tromsø Hospital Pharmacy, Tromsø, Norway

**Keywords:** Analgesics, Chronic pain, Pharmacoepidemiology, Cohort, Pain sensitivity, Cold pressor test, QST, Opioid-induced hyperalgesia

## Abstract

**Background:**

Increased pain sensitivity is a putative risk factor for chronic pain and consequently for analgesic use. Conversely, analgesic use may be a cause of increased pain sensitivity, e.g., through opioid-induced hyperalgesia. We aimed to study the association between pain sensitivity and analgesic use in a general population, and to test the hypothesis that increased baseline pain sensitivity is a risk factor for future persistent analgesic use.

**Methods:**

The Tromsø Study (2007–08), a population-based health study, was linked with eight years of prescription data from the Norwegian Prescription Database. The cold pressor test was completed in 10,486 participants aged 30+ years, and we used cold pressor endurance time as a proxy measure of pain sensitivity. Cross-sectional associations with different measures of analgesic use were assessed. Furthermore, a cohort of 9,657 persons was followed for 4.5 years.

**Results:**

In the cross-sectional analysis, increased pain sensitivity was associated with analgesic use; regular users of opioids alone were more pain sensitive than regular users of non-opioid analgesics. Increased baseline pain sensitivity was a risk factor for persistent analgesic use, i.e., using non-steroidal anti-inflammatory drugs, paracetamol, or opioids for ≥ 90 days and proportion-of-days-covered ≥ 40% (HR = 1.22, 95% CI 1.06-1.40), although not statistical significant after confounder adjustment.

**Conclusions:**

Increased pain sensitivity was associated with analgesic use in general, and reduced pain tolerance was found for both opioid and non-opioid analgesic users. The data suggest that hyperalgesia is an effect of analgesics, whereas pain tolerance has little impact on future analgesic use.

**Electronic supplementary material:**

The online version of this article (doi:10.1186/s40360-017-0149-2) contains supplementary material, which is available to authorized users.

## Background

The efficacy of analgesics varies according to type of analgesic and type of pain; in a review paper by Oertel and Lötsch, opioids showed the most positive evidence for the treatment of various kinds of clinical pain, followed by non-steroidal anti-inflammatory drugs (NSAIDs) [1]. Long-term analgesic use and use in chronic pain have limited evidence of efficacy or effectiveness [2–7]. However, as many clinical studies on analgesics report average differences in pain between groups, treatment responders may be missed or the treatment effect within certain subgroups may be attenuated [4, 8, 9]. In this regard, there has been a growing interest in mechanism-based treatment of pain, i.e., finding and treating potential treatment responders on the basis of the pathophysiological pain mechanisms involved [8, 9], and whether experimental pain tests can be used to predict if a patient would respond to an analgesic [10, 11]. We have previously shown that the prevalence of persistent prescription (Rx) analgesic use is only ten percent among those reporting chronic pain, and we suggest that this group may represent those who benefit from long-term treatment and have not discontinued due to adverse effects [12]. Moore et al. point out that if the patient responds to treatment, the benefit is often long-lasting [4]. However, persistent use of analgesics may not necessarily reflect an adequate and prolonged treatment effect but may also be due to irrational use, or, sometimes for the opioids, due to drug abuse.

The potential causal pathways between pain sensitivity, chronic pain and analgesic use are not clear. Edwards proposes that increased basal pain sensitivity is a “diathesis for chronic pain” [13]. On the contrary, there is evidence from a study on tension-type headache that continued peripheral nociceptive activity causes central sensitization and increased pain sensitivity [14]. Furthermore, according to Edwards, persons with increased pain sensitivity may have reduced endogenous pain inhibition and analgesics may work less effective among these subjects [13]. Finally, a growing body of evidence shows that opioid use may paradoxically increase pain sensitivity through opioid-induced hyperalgesia (OIH) [15–18].

To our knowledge, no study of the association between pain sensitivity and analgesic use in a general population exists. The aims of this study were to 1) assess the association between pain sensitivity, measured by the cold pressor test (CPT), and analgesic use, including persistent analgesic use, and 2) to explore if increased baseline pain sensitivity is a risk factor for future persistent analgesic use. To achieve these aims, we linked the largest pain sensitivity study to date, the Tromsø 6 study, with eight years of individual-level dispensing data from the Norwegian Prescription Database (NorPD). This study is based on methods and study design as previously reported by us [12].

## Methods

### Study population

The Tromsø Study is a population-based, prospective health study carried out in the municipality of Tromsø, Norway, and includes a representative sample of the general population [19]. The current study includes participants aged 30–87 years (*n* = 10,486), from the sixth wave (Tromsø 6) conducted in 2007–08, who underwent CPT. The data collection and sampling procedure for Tromsø 6 have been extensively described previously [19]. Relevant variables from Tromsø 6, collected at attendance, comprise self-reported data on chronic pain, analgesic use, sociodemographic and comorbid factors gathered through two written questionnaires, in addition to CPT, as described in more details below. A first questionnaire was sent out by post with the invitation letter approximately two weeks before attendance. A second questionnaire, containing follow-up questions, was given at attendance, and it could be either filled out at the location or sent in afterwards by post [12].

We linked the Tromsø 6 study to NorPD. NorPD is a national registry of all prescriptions dispensed to individual patients in Norwegian pharmacies, i.e., covering the entire Norwegian population [20]. NorPD does not register drugs dispensed in hospitals, nursing homes, or directly from the physician, or non-prescription (OTC) drugs. Included NorPD variables in this study were date of dispensing, Anatomical Therapeutic Chemical (ATC) code, defined daily dose (DDD), and the de-identified serial number used for record-linkage. The ATC system classifies drugs on the basis of therapeutic, pharmacological, and chemical properties. The DDD represents the assumed average maintenance dose in monotherapy for the main indication in adults [21].

### Cold pressor test (CPT)

The planned sample to undergo CPT included all participants attending Tromsø 6 (*n* = 12,984) [19]. However, due to capacity restrictions some participants were not tested [22]. Hence, subjects < 60 years were prioritized, due to the lower sampling rate for these age cohorts.

In the CPT, participants immersed their hand and wrist in circulating cold water and held it there as long as they could, up to a maximum of 106 s [22, 23]. Participants rated their pain intensity on a 0–10 numerical rating scale (NRS) after 4 s and every 9th s thereafter. Endurance time, i.e., cold pain tolerance time, was recorded on hand-withdrawal.

The CPT equipment consisted of a Julabo FP40HE water bath (Julabo Labortechnik GmbH, Germany) from which water was pumped to an external 13-L container, with a constant temperature of 3.0 °C and circulation speed of 22 L/min.

### Definition of analgesic use

Our main variable of interest was persistent prescription analgesic use, which was calculated on the basis of the dispensing data from NorPD. Analgesics included were: a) Non-steroidal anti-inflammatory drugs (NSAIDs, ATC group M01A, excluding M01AX05 glucosamine), b) opioids (N02A), or c) other analgesics and antipyretics (N02B), which in practice consisted of paracetamol (acetaminophen). Atypical/adjuvant analgesics constituted only a small fraction of the total prescription volume and were not included due to ambiguous indication for use, e.g., treatment of depression or pain [12]. Briefly, we collapsed the aforementioned analgesic groups into a combined measure of analgesic use and identified persistent treatment episodes of analgesics. Prescriptions dispensed within 180 days of one another belonged to the same treatment episode; subjects were defined as being under persistent use if the treatment episode lasted 90 days or more and the proportion-of-days-covered (PDC) with analgesics was 40% or higher [12]. In this context, the PDC was calculated on the basis of the DDD and reflects a measure of intensity of use. As examples of persistent analgesic use, this definition will roughly correspond to an annual consumption of at least 440 tablets of paracetamol 1 g or 300 tablets of ibuprofen 600 mg.

Moreover, participants of the Tromsø 6 study reported any use of OTC and Rx analgesics, and all drugs used regularly last four weeks [24], in addition to any analgesic use within 24 h before the CPT.

A more detailed description of the analgesic use measures can be found in an Additional file [see Additional file 1].

### Confounding variables

Baseline chronic pain at attendance was defined as persistent or constantly recurring pain lasting three months or more [12]. Age was categorized into 30–44, 45–59, 60–74, and ≥ 75 years, due to a non-linear association with both cold pain tolerance and persistent analgesic use [12]. Education was divided into primary/secondary school (≤9 years), upper secondary education (10–12 years), college/university (less than four years), and college/university (four years or more). Physical activity and psychological distress (Hopkins Symptoms Checklist (HSCL-10) score > 1.85) were also considered [12]. However, as physical activity was not associated with pain sensitivity at the cross-sectional level, and psychological distress was not statistically significantly associated with persistent analgesic use in the previous study [12], these variables were left out of the regression models.

### Study design

The study period was three years (1095 days) before to five years (1825 days) after the attendance date, i.e., a total study period of eight years.

This study has two parts: A cross-sectional part to study the association between cold pressor endurance time and different measures of analgesic use, including persistent analgesic use, derived from the dispensing data, and self-reported analgesic use in the four weeks preceding attendance. If a persistent treatment episode overlapped the attendance date, the subject was defined as a prevalent persistent analgesic user.

The second part consisted of a prospective analysis of the association of baseline pain sensitivity with future persistent analgesic use, based on the dispensing data. We constructed a new cohort by excluding 829 subjects who were prevalent or previous persistent analgesic users within the three years preceding attendance (*n* = 9,657). The start date of the first persistent treatment episode was defined as the event date, while death and the end of follow-up were censoring dates. The follow-up time was 4.5 years (1,640 days).

### Statistical analysis

In the cross-sectional analysis, we used survival analysis entering endurance time as the survival time, hand-withdrawal as the event and reaching the limit at 106 s as censoring, as in several previous studies [22, 23, 25]. The measures of analgesic use were entered as exposure variables.

In the prospective analysis, we used endurance time as the exposure variable, dichotomized into <106 s and 106 s, i.e., those who did and did not withdraw their hand in the CPT, respectively. Due to the severely right-censored nature of this variable [23] we did not find it appropriate to model it as a continuous variable, or based on percentiles [26]. This empirically based choice was also motivated by considerations including the proportional hazard (PH) assumption and power.

We used Cox PH regression with Breslow method for ties in both the cross-sectional and prospective analyses. The PH assumption was assessed graphically and by test of scaled Schoenfeld residuals. The PH assumption was not violated in the prospective analysis, or for the main variable, persistent analgesic use, at the cross-sectional level. However, as self-reported OTC/Rx use, analgesic use last 24 h, sex, age, education, and chronic pain seemed to violate the PH assumption at the cross-sectional level, we also employed extended Cox models including possible time varying effects of the covariates. As the results and interpretation largely were the same, we find it sufficient to report the ordinary Cox regression. Associations are reported as hazard ratios (HR) with 95% confidence intervals (CI).

In the cross-sectional analysis, a HR > 1 implies increased pain sensitivity, i.e., reduced pain tolerance, compared to the reference group [22].

In the prospective analysis, a HR > 1 implies increased risk of future persistent analgesic use among those who withdrew their hand compared to those who endured the whole CPT.

A *p* value < .05 was considered statistical significant. The proportion of missing in the various regression models was ≤ 4%, and ≤ 1% in the models including persistent analgesic use, and we deemed multiple imputation as unnecessary. All analyses were performed in Stata 14 (Stata Corp, College Station, TX).

## Results

### Study population and prospective study cohort

The study population comprised of 10,486 men and women in the age range 30–87 years who completed the CPT. Sixty-eight per cent (*n* = 7,157) reached the CPT endurance time maximum of 106 s. Characteristics of the total study population and the prospective study cohort are shown in Table 1.

**Table 1:** Descriptive statistics of the study population stratified on persistent analgesic use and in total (*n* = 10,486), and among the prospective study cohort at baseline (*n* = 9,657)

		Not persistent analgesic users		Persistent analgesic users		*Total study population*		*Study cohort at baseline*	
Age, y, median, IQR		58	45–65	59	47–67	58	45–65	58	45–65
Women, % *n*		51.2	5,151	59.7	253	51.5	5,404	51.0	4,929
Education, % *n*	Primary/secondary school	26.0	2,584	36.2	152	26.4	2,736	25.8	2,463
	Upper secondary education	33.9	3,374	38.3	161	34.1	3,535	33.6	3,211
	College/university (less than four years)	18.6	1,852	12.9	54	18.4	1,906	18.8	1,794
	College/university (four years or more)	21.6	2,146	12.6	53	21.2	2,199	21.9	2,091
Physical activity^a^, % *n*	Never or less than once a week	21.0	2,068	28.5	117	21.3	2,185	21.0	1,985
	Once a week	20.3	1,996	21.2	87	20.3	2,083	20.5	1,934
	2-3 times a week	39.3	3,866	32.8	135	39.1	4,001	39.2	3,702
	Approximately every day	19.4	1,903	17.5	72	19.3	1,975	19.3	1,824
Psychological distress, % *n*		7.2	694	19.5	79	7.7	773	7.1	659
Chronic pain, % *n*		32.1	3,226	82.3	348	34.1	3,574	31.2	3,004
Cold pressor test	Cold endurance time (s), median, IQR	106	71–106	106	46–106	106	70–106	106	71–106

*IQR* interquartile range, *NRS* numerical rating scale

^a^Frequency of exercise

Of the study population, 829 persons were prevalent or previous persistent analgesic users, creating a new cohort of 9,657 persons for prospective analysis. Within the follow-up (41,311 person-years, median 4.5 years = 1,640 days), the number of incident cases of persistent analgesic use, i.e., the first episode of persistent analgesic use, was 836.

### Association of pain sensitivity with analgesic use at the cross-sectional level

**Fig. 1:**
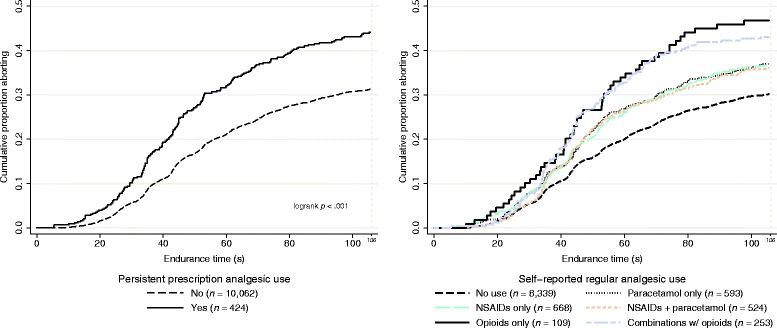
Cold pressor tolerance and persistent prescription analgesic use (left) and self-reported regular analgesic use (right). The y-axis represents the cumulative proportion withdrawing their hand in the cold pressor test. Comparison with regular users of opioids alone (Wald test): paracetamol alone (*p* = .044), NSAIDs alone (*p* = .036), users of both paracetamol and NSAIDs (*p* = .029), and users of both opioids and non-opioids (*p* = .56)

All measures of analgesic use were consistently associated with increased pain sensitivity, i.e., reduced pain tolerance (HR > 1), in crude analyses (Table 2 & Fig. 1). The associations generally remained statistical significant after adjustment for age, sex, education, and chronic pain.

**Table 2:** Associations between pain sensitivity and different measures of analgesic use (n = 10,486). Cross-sectional analysis

		Prevalence		Crude		Model A		Model B	
		*n*	%	HR^a^	95% CI	HR	95% CI	HR	95% CI
Persistent Rx analgesic use^b^	No	10,062	96.0	1	Ref	1	Ref	1	Ref
	Yes	424	4.0	1.58	1.37–1.83	1.45	1.25–1.68	1.33	1.14–1.55
*Self-reported analgesic use*									
Any use last four weeks	No use	5,461	53.8	1	Ref	1	Ref	1	Ref
	OTC only	3,292	32.4	1.32	1.22–1.42	1.14	1.05–1.23	1.11	1.03–1.21
	Rx only	495	4.9	1.47	1.26–1.72	1.28	1.10–1.50	1.20	1.02–1.41
	Both OTC and Rx	909	8.9	1.59	1.42–1.79	1.30	1.15–1.46	1.20	1.06–1.36
Regular use last four weeks^c^	No use	8,339	79.5	1	Ref	1	Ref	1	Ref
	Paracetamol only^d^	593	5.7	1.30	1.13–1.49	1.07	0.93–1.23	1.03	0.90–1.19
	NSAIDs only	668	6.4	1.28	1.13–1.46	1.16	1.02–1.33	1.11	0.97–1.27
	NSAIDs + paracetamol^d^	524	5.0	1.26	1.08–1.46	1.06	0.91–1.23	0.99	0.85–1.16
	Opioids only	109	1.0	1.77	1.34–2.34	1.49	1.12–1.98	1.36	1.02–1.81
	Combinations w/opioids	253	2.4	1.60	1.32–1.94	1.42	1.17–1.72	1.29	1.06–1.57
Last 24 hours^e^	No	9,502	92.4	1	Ref	1	Ref	1	Ref
	Yes	776	7.6	1.67	1.50–1.87	1.48	1.32–1.66	1.40	1.25–1.57

*HR* hazard ratio, *CI* confidence interval, *OTC* non-prescription, *Rx* prescription, *NSAIDs* non-steroidal anti-inflammatory drugs

Model A: Adjusted for age, sex and education. Missing in the model including persistent analgesic use: *n* = 110 (1.05%)

Model B: Same as A but including chronic pain. Missing in the model including persistent analgesic use: *n* = 123 (1.17%)

^a^HR > 1 implies increased pain sensitivity, i.e., reduced cold pain tolerance, compared to the reference group

^b^Use of NSAIDs, paracetamol or opioids for ≥ 90 days and with a proportion-of-days-covered ≥ 40%. In the study period, the persistent treatment episodes consisted on average of 39.3% NSAID, 44.0% opioid and 16.7% paracetamol prescriptions

^c^Only the “classical” analgesic groups NSAIDs, paracetamol or opioids are counted here, i.e. adjuvant/atypical analgesics are not included

^d^“Paracetamol” includes the Anatomical Therapeutic Chemical group N02B “Other analgesics and antipyretics”, and consists almost exclusively of paracetamol use but also minor use of phenazone-caffeine or aspirin (acetylsalicylic acid) (see [24])

^e^Use of any analgesics within the 24 h prior to the cold pressor test

Those who reported regular opioid use seemed more pain sensitive than those reporting regular use of non-opioid analgesics (Table 2 & Fig. 1); regular users of opioids alone were more pain sensitive than users of paracetamol alone (*p* = .044), NSAIDs alone (*p* = .036), or users of both paracetamol and NSAIDs (*p* = .029) in the crude analysis. However, those who combined opioids and non-opiods were not statistically significantly different from users of opioids alone, paracetamol alone, or NSAIDs alone. This is visualized in the figure (Fig. 1., right): no use at the bottom, use of non-opioid analgesics in the middle, and use of opioids, either alone or in combination with non-opioid analgesics, on the top. Here “paracetamol” consists almost exclusively of paracetamol use but also minor use of phenazone-caffeine or aspirin (acetylsalicylic acid) (see [24]). The opioid groups remained statistical significant also after adjustment for potential confounders (Table 2).

### Association of baseline pain sensitivity with future persistent analgesic use

Increased baseline pain sensitivity was associated with an increased risk of future persistent analgesic use in the crude analysis (HR = 1.22, 95% CI 1.06-1.40) (Table 3). The point estimates remained positive but the strength of the association was diminished and non-significant after adjustment for age, sex, education, and chronic pain at baseline.

**Table 3:** Baseline pain sensitivity and the risk of future persistent analgesic use within the 4.5 years of follow-up (*n* = 9,657). Prospective analysis

	Crude	*n* = 9,657	Model A	*n* = 9,559	Model B	*n* = 9,548
	HR	95% CI	HR	95% CI	HR	95% CI
Withdrew hand^a^	1.22	1.06–1.40	1.13	0.97–1.30	1.09	0.94–1.26
Did not withdraw hand	1	Ref	1	Ref	1	Ref

*HR* hazard ratio, *CI* confidence interval

Model A: Adjusted for age, sex and education

Model B: Same as A but including chronic pain

Persistent analgesic use: Use of NSAIDs, paracetamol or opioids for ≥ 90 days and with a proportion-of-days-covered ≥ 40%. In the study period, the persistent treatment episodes consisted on average of 39.3% NSAID, 44.0% opioid and 16.7% paracetamol prescriptions

^a^This group withdrew their hand in the cold pressor test. The reference group consists of those who endured the entire test of 106 s. Those who withdrew their hand are assumed less cold pain tolerant, i.e., more pain sensitive

## Discussion

### Main findings

In this large population-based linkage study, the main findings were that increased pain sensitivity is associated with analgesic use at the cross-sectional level, regular users of opioids alone were more pain sensitive than regular users of non-opioid analgesics, i.e., NSAIDs and paracetamol, and that increased baseline pain sensitivity was a risk factor for future persistent analgesic use in crude analysis, but not in multivariable analyses. This is to our knowledge the first report on the association between pain sensitivity and analgesic use in a general population.

### Analgesic use causing increased pain sensitivity

Long-term analgesic use may cause a paradoxical increase in pain, e.g., through medication-overuse headache [27] or OIH. OIH can be described as an increase in pain sensitivity, which is not accounted for by withdrawal symptoms [15] or a deterioration of the pain-causing disease [18], but the clinical relevance is somewhat contentious [16]. In previous studies, OIH has been defined by a reduced pain tolerance in opioid users [15, 16].

The literature on opioid use is extensive but less attention has been given to non-opioid analgesics. In a cross-sectional study in pain patients by Lötsch et al., the use of opioids was associated with lower pain scores compared to non-users [28]. Surprisingly, use of non-opioid antipyretic analgesics alone, including NSAIDs and paracetamol, was associated with higher pain scores, leading the authors to hypothesize that long-term cyclooxygenase inhibition may have counter-analgesic effects [28]. Although this finding needs confirmation from prospective studies, this opens the possibility that paradoxically increased levels of pain are not confined to the use of opioids.

Our results do indeed suggest that regular users of opioids alone are more pain sensitive compared to regular users of NSAIDs/paracetamol, and that the NSAID or paracetamol users have increased pain sensitivity compared to non-users of analgesics. This could suggest the presence of OIH and possibly counter-analgesic effects of NSAIDs. However, as Edwards et al. point out, preexisting hyperalgesia in chronic pain patients may make it challenging to identify an independent effect of OIH, particularly at the cross-sectional level [29]. Furthermore, the pattern of increased pain sensitivity among analgesic users was consistent for different measures of analgesic use (e.g., prescription status, analgesic type, frequency of use) suggesting severity of the underlying pain as the main explanation.

### Pain sensitivity and the effectiveness of analgesics

It may seem logical that subjects with increased inherent pain sensitivity are more likely to use analgesics. However, Edwards suggest that the effectiveness of analgesics is reduced in a state of increased pain sensitivity [13]. Increased pain sensitivity may be explained by a “disruption of endogenous pain inhibitory processes” [30], and the mechanism of action of analgesics is by “recruiting” these pain inhibitory processes [13]. Previous studies have reported that a large proportion of opioid users continue to report severe chronic pain [31, 32]. Indeed, in our study population over one third of those reporting chronic pain reported usual pain intensity as severe (NRS ≥ 7) despite using analgesics persistently, while pain sensitivity increased with increasing chronic pain intensity (data not shown). As previously suggested, these traits may be associated with involvement of central pain mechanisms [31]. Classical analgesics, including the opioids, are less effective in pain phenotypes with documented change in central pain mechanisms [1, 31, 33]. Based on this and the assumption that persistent analgesic users are a sub group of treatment responders, i.e., where analgesics are effective, one would expect that increased baseline pain sensitivity would *not* increase the risk of using classical analgesics persistently in the future. Indeed, no association was found when potential confounders were included. However, cautious interpretation is advised as the null finding may also be explained by insufficient power or follow-up time.

### Potential causal pathways

**Fig. 2:**
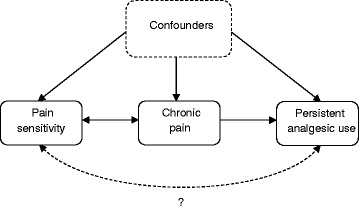
Potential causal relationships between pain sensitivity, chronic pain, and persistent analgesic use

The associations between pain sensitivity, chronic pain and persistent analgesic use are complex, both pharmacologically/physiologically and statistically (i.e., whether to adjust for chronic pain or not). Potential causal pathways are illustrated in Fig. 2. Increased pain sensitivity may be both a cause and a consequence of chronic pain [13, 14], or there may be a common pathophysiological mechanism. Persistent analgesic use may cause increased pain sensitivity/hyperalgesia. However, in a prospective design where pain sensitivity is measured before the start of persistent analgesic use, this is less likely, but may explain some of the findings in the cross-sectional analysis. Finally, measured or unmeasured confounders may lead to spurious findings due to incomplete adjustment and residual confounding. Nevertheless, given these caveats we propose several possible explanations or hypotheses: a) increased pain sensitivity among analgesic users is due to hyperalgesia being associated with the severity of the underlying pain condition, b) analgesic use do not restore the endogenous pain inhibitory systems to a healthy/normal state, c) the effectiveness of classical analgesics is reduced in a state of increased pain sensitivity, possibly due to more central pain mechanisms [13], d) increased pain sensitivity is a consequence of pharmacological treatment of clinical pain, first and foremost through OIH but the role of long-term NSAID use should be explored.

### Study strengths and limitations

The major strengths of this study include the large population-based sample linked with individual-level “gold standard” drug data from a national prescription database, self-reported data on analgesic use, chronic pain, and sociodemographic and comorbidity variables, as well as measurements of pain sensitivity.

Experimental pain tests, like the CPT, represent a proxy measure of pain, as they measure a “psychophysical or bioresponse to nociceptive stimulation” [1]. However, the pain induced by the CPT is a deep, tonic, aching pain, believed to be more clinically relevant than pain threshold tests [22, 34, 35], and which may be more suitable in studies on the effects of analgesics [10, 11]. The response to the CPT has been shown to be reproducible [35], with the CPT procedure used in Tromsø 6 demonstrating a test-retest correlation of α = 0.82 [36].

We did not include or adjust for different chronic pain causes or somatic conditions in the analysis, e.g., migraine, rheumatoid arthritis, or neuropathic pain. The associations between pain sensitivity and analgesic use may differ in different chronic pain states.

Self-reported analgesic use is subject to recall bias and is probably underestimated [24]. The prescription registry contains data on *dispensed* drugs and as such represents only a proxy of use. Further methodological discussion of the measures of analgesic use is presented elsewhere [12, 24].

In terms of external validity, dissimilarities in chronic pain prevalence, pain sensitivity, and analgesic utilization between countries and populations may make it challenging to extrapolate our results [12]. Nevertheless, we believe, as previously stated, that our study population represents a typical Northern European, predominantly white, urban population [19].

## Conclusions

Increased pain sensitivity was associated with analgesic use in general at the cross-sectional level, with regular users of opioids alone being more pain sensitive than regular non-opioid users. Though increased pain sensitivity was associated with future persistent analgesic use, this association was weak, and non-significant after controlling for confounders. The data therefore suggest that hyperalgesia is an effect of analgesics, whereas pain tolerance has little impact on future analgesic use. The potential causal mechanisms are, however, hidden in the black box for now. Prospective studies, with several points of measurements of pain, pain sensitivity, and confounders, are needed to elucidate potential causal pathways between pain sensitivity, chronic pain, and analgesic use.

## Additional file

Additional file 1: In-depth description of the definitions of analgesic use. (DOCX 19 kb)

## Abbreviations

ATC: Anatomical therapeutic chemical classification system
CI: Confidence interval
CPT: Cold pressor test
DDD: Defined daily dose
HR: Hazard ratio
HSCL-10: Hopkins symptoms checklist 10-item version
NorPD: Norwegian Prescription Database
NRS: Numerical rating scale
NSAIDs: Non-steroidal anti-inflammatory drugs
OIH: Opioid-induced hyperalgesia
OTC: Non-prescription
PDC: Proportion-of-days-covered
PH: Proportional hazard
Rx: Prescription
