# Supplementary material for: Pain sensitivity and analgesic use among 10,486 adults: the Tromsø study
Source: BMC Pharmacol Toxicol. 2017 Jun 9;18:45. doi: 10.1186/s40360-017-0149-2 (PMC5466805; doi:10.1186/s40360-017-0149-2)
Supplement: Additional file 1: — In-depth description of the definitions of analgesic use. (DOCX 19 kb) [file 40360_2017_149_MOESM1_ESM.docx]

Additional file

# Definition of analgesic use

The following contains a more detailed description of how the analgesic use measures were defined. The descriptions are cited verbatim from previous papers (citations/references to figures etc. within the texts are removed) [1, 2].

Self-reported analgesic use, from Samuelsen, Slørdal, Mathisen and Eggen [1]:

*“Analgesic use was assessed through questionnaire based on the question “How often have you used painkillers [with]/[without] prescription during the last four weeks?”. Analgesic users were defined as persons reporting any use. This variable was recoded into use of OTC analgesics only (“OTC”), use of prescribed analgesics only (“Rx”) and use of concomitantly OTC and prescribed analgesics (“OTC + Rx”). Participants in Tromsø 6 also reported drugs used regularly the preceding four weeks; this was coded according to the Anatomical Therapeutic Chemical (ATC) classification system version 2007 (www.​whocc.​no). Analgesics were defined as belonging to ATC groups N02B (other antipyretic and analgesic drugs), N02A (opioids) and M01A (NSAIDs, excluding glucosamine).”*

Persistent analgesic use, from Samuelsen, Svendsen, Wilsgaard, Stubhaug, Nielsen and Eggen [2]:

*“Analgesics included were: a) NSAIDs (ATC group M01A, excluding M01AX05 glucosamine), b) opioids (N02A), or c) other analgesics and antipyretics (N02B). As the latter group consisted of 99.8% paracetamol prescriptions, it will only be referred to as “paracetamol”. We combined the different analgesic groups into a joint measure of analgesic use in order to capture the combination users and those switching analgesic groups. Atypical/adjuvant analgesics were only included in supplementary analyses due to equivocal indications for use. Furthermore, participants reported use of OTC analgesics last four weeks.*

*We adapted the method from Von Korff et al. and Poluzzi et al. and defined analgesic treatment episodes; analgesic prescriptions belonged to the same treatment episode if the time gap between the prescriptions was ≤ 180 days. Consequently, a treatment episode started with no analgesic prescriptions in the prior 180 days and an analgesic prescription within the next 180 days. The prescription with no subsequent prescriptions in the following 180 days was defined as the last prescription in that treatment episode. If several prescriptions were dispensed on the same date, the DDDs were summed. The duration of the last prescription was calculated as the number of DDDs dispensed divided by the average daily dose, limited to maximum 180 days. The duration of a treatment episode was the number of days between the first and last prescription plus the treatment duration of the last prescription.*

*Proportion-of-days-covered (PDC) is a measure of the proportion of days within a time interval with a*n available supply of drugs*. PDC was calculated as the cumulative number of DDDs within a treatment episode divided by the treatm*ent duration of the episode*. For a treatment episode to be persistent the PDC had to be ≥ 40% and the duration of the episode had to be ≥ 90 days (see sensitivity analysis in Supplement). The cut-off of ≥ 90 days was partly chosen to reflect the Norwegian reimbursement system, whereby reimbursement is given when the total treatment duration is* at least three months per year. *”*

# References

1 Samuelsen PJ, Slørdal L, Mathisen UD, Eggen AE: **Analgesic use in a Norwegian general population: change over time and high-risk use - The Tromsø Study**. *BMC Pharmacol Toxicol* 2015, **16**(1):16.

2 Samuelsen PJ, Svendsen K, Wilsgaard T, Stubhaug A, Nielsen CS, Eggen AE: **Persistent analgesic use and the association with chronic pain and other risk factors in the population-a longitudinal study from the Tromso Study and the Norwegian Prescription Database**. *Eur J Clin Pharmacol* 2016, **72**(8):977-985.
